# Supplementary material for: The interactome of intact mitochondria by cross-linking mass spectrometry provides evidence for coexisting respiratory supercomplexes
Source: Mol Cell Proteomics. 2017 Dec 8;17(2):216–32. doi: 10.1074/mcp.RA117.000470 (PMC5795388; doi:10.1074/mcp.RA117.000470)
Supplement: Supplemental Data [file supp_RA117.000470_133922_0_supp_23978_fzffwf.zip › spectra_annotation/mito_spectra_annotation/134-2-7-1-3-1.pdf]

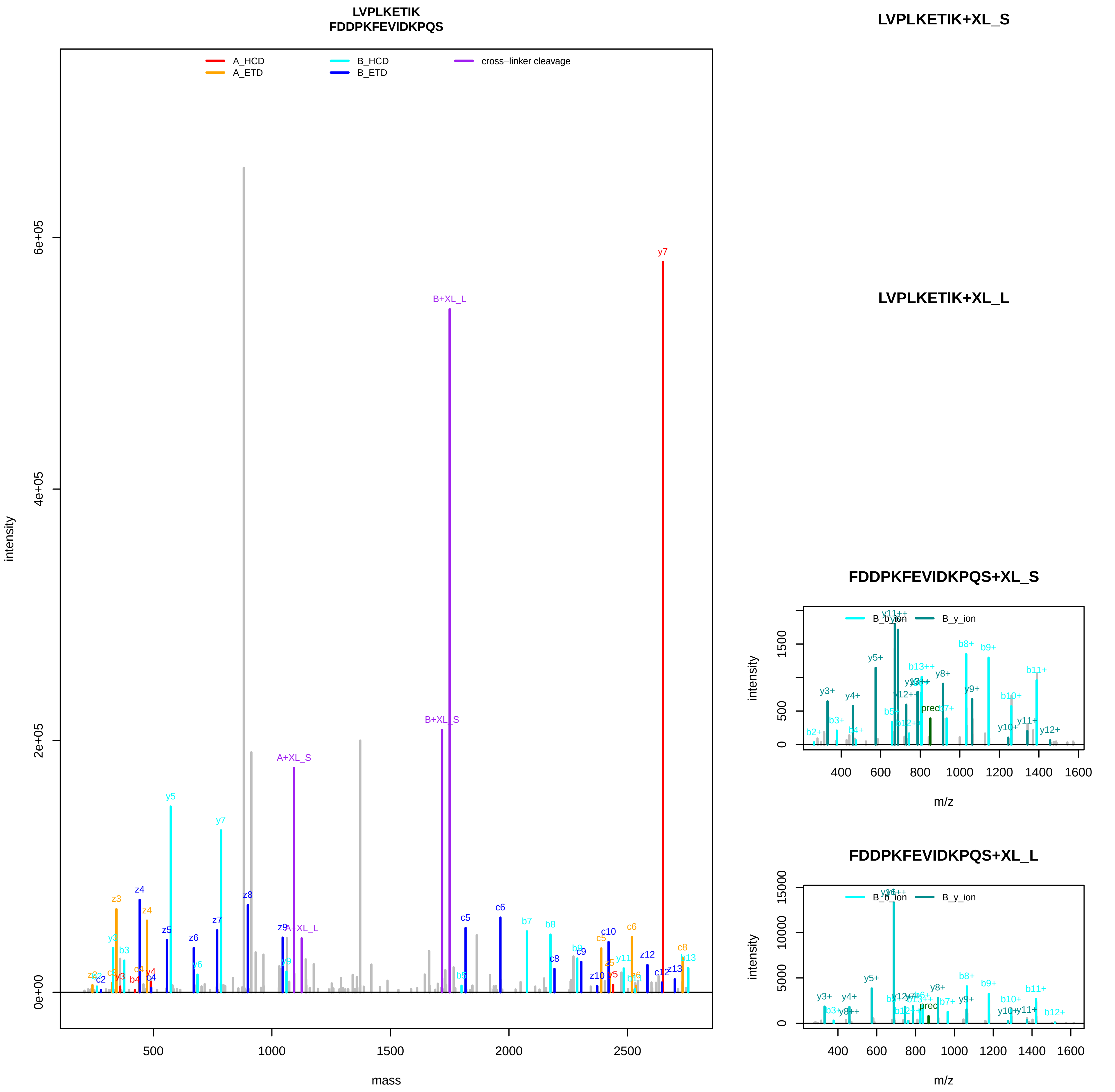
LVPLKETIK
FDDPKFEVIDKPQS
A_HCD
A_ETD
B_HCD
B_ETD
cross−linker cleavage
B+XL_L
B+XL_S
A+XL_S
A+XL_L
intensity
0e+00
2e+05
4e+05
6e+05
mass
500
1000
1500
2000
2500
LVPLKETIK+XL_S
LVPLKETIK+XL_L
FDDPKFEVIDKPQS+XL_S
B_b_ion
B_y_ion
intensity
0
500
1500
m/z
400
600
800
1000
1200
1400
1600
FDDPKFEVIDKPQS+XL_L
B_b_ion
B_y_ion
intensity
0
5000
10000
15000
m/z
400
600
800
1000
1200
1400
1600
